# ﻿A revision of the genus *Cistanche* (Orobanchaceae) in Israel, and considerations for its taxonomic circumscription in the Middle East

**DOI:** 10.3897/phytokeys.260.158426

**Published:** 2025-08-01

**Authors:** Dar Ben-Natan, Chris Thorogood

**Affiliations:** 1 Deshe open landscape institute, Steinhardt Museum of natural history, Tel-Aviv University, Klausner St. 12, Tel Aviv-Yafo, Israel Deshe Open Landscape Institute, Steinhardt Museum of Natural History, Tel Aviv University Tel Aviv Israel; 2 University of Oxford Botanic Garden, Rose Lane, Oxford, OX1 4AZ, UK University of Oxford Botanic Garden Oxford United Kingdom; 3 Department of Biology, University of Oxford, South Parks Road, Oxford, OX1 3RB, UK University of Oxford Oxford United Kingdom

**Keywords:** Holoparasite, new species, nomenclature, parasitic plant, taxonomy

## Abstract

The genus *Cistanche* is the subject of considerable taxonomic confusion due to reduced morphology, poor preservation in herbaria, and misidentification. Here we re-examine the *Cistanche* of Israel, a region that falls within the broader Middle Eastern centre of diversity for the genus, but has been neglected from systematic attention. In our reconsideration we address taxonomic confusion in the region and confirm the presence of *C.tubulosa* and *C.violacea*, report for the first time the occurrence of *C.laxiflora* plus a separate entity we refer to as *C.tinctoria*, and describe a new species, *C.mimii*. We observe considerable phenotypic plasticity hitherto unreported, for example fruit valve number. Together, our findings suggest that species circumscription and diversity are still poorly understood and require careful examination in the field at a local scale. We provide a key for all *Cistanche* species in the region, along with an assessment of their ecology and conservation considerations. Finally, we consider our findings within the broader context systematics in this complicated genus.

## ﻿Introduction

The genus *Cistanche* Hoffmanns. & Link (family Orobanchaceae) is widely distributed across semi-arid and arid regions of the Old World from Macaronesia, western Africa, and the Mediterranean Basin, to central and eastern Asia. Most of the 20–30 accepted species grow on dunes, alluvial plains and in deserts where they are parasitic on the roots of xerophytic and halophytic shrubs, especially those in the family Amaranthaceae, and also the Tamaricaceae. Despite the widespread use of *Cistanche* in traditional herbal medicine (Wang et al. 2019; Thorogood et al. 2021), and the conservation concern of traded species, the genus remains poorly understood, and like other holoparasitic (non-photosynthetic) genera in the Orobanchaceae, is the subject of significant taxonomic confusion. This confusion stems from a combination of a reduction in morphological features (a lack of developed leaves, for example), poor preservation in herbaria, conflicting national accounts of species’ ranges and a lack of sampling across much of the genus’ range. Given the lack of vegetative features, diagnostic emphasis has been placed on inflorescence and flower characteristics including floral bracts and bracteole morphology and corolla colour, shape and indumentum (Beck-Mannagetta 1930; Ataei et al. 2020). However, many of these characteristics (especially colour), preserve inadequately in herbaria, and calyx morphology is highly variable in the field. Therefore, a combined approach that examines morphology, ecology and phylogenetics is required to tease apart species, as suggested for the related (and similarly problematic) genus *Orobanche* L. (Thorogood and Rumsey 2020; Thorogood and Rumsey 2021).

The last monograph of the genus *Cistanche*, published nearly a century ago (Beck-Mannagetta 1930) recognised four taxonomic sections, based on calyx and bracteole characteristics: (i) C.sect.Cistanchella containing *C.ridgewayana* Aitch. and Hemsl. (1888); (ii) C.sect.Subcistanche containing *C.sinensis*Beck-Mannagetta (1930); (iii) C.sect.Heterocalyx, containing three species: *C.fissa* (CA Mey.) Beck-Mannagetta (1897), *C.ambigua* (Bunge) Beck-Mannagetta (1930) and *C.rosea*Baker (1895); and (iv) C.sect.Eucistanche containing all remaining species (Table 1). Subsequent treatments have added additional species to this traditional framework, but species limits in the genus remain confused. The first phylogenetic assessment of the genus using plastid and nuclear markers identified four geographically differentiated clades: three from East Asia, Northwest Africa, Southwest Asia, and one ‘widespread clade’ (Ataei et al. 2020). Only the East Asian clade corresponded with a previously recognised section, refuting former understanding of the taxonomy of the genus. Our recent phylogenetic work including samples from Europe, Israel and the wider Middle East, North Africa and China (in prep.) corroborates the presence of these clades, but highlights the application of names to polyphyletic entities, emphasizing the need for a firmer grasp of species diversity at a local scale. Therefore, ahead of publishing this phylogeny, here we seek to clarify the taxonomy of Israeli plants in particular – an area that is likely to fall within the Middle Eastern centre of diversity of the genus, but which has been neglected from previous studies. Taxonomic resolution at a local scale, including careful observation of plants in the field, will enable a much-needed, robust circumscription of the genus at a global level, and is necessary in advance of a broadscale phylogeny to apply nomenclature objectively.

**Table 1.** Diagnostic characters for species traditionally placed in sect. Eucistanche. Details for *C.laxiflora*, *C.violacea*, *C.tinctoria* and *C.tubulosa* are based on observations of living and dried material (see *Specimens Examined*). Details for the other taxa were adapted from Beck-Mannagetta (1930).

Species	Corolla colour	Bract margins	Folds in corolla throat	Number of capsule valves
*C.laxiflora*	Tube white, limb lilac or very light violet, pale.	Entire, non-scarious, glabrous. White, grey to off-white, or pale lilac.	Very shallow, inconspicuous folds, yellow.	2–4. Usually 3–4 (in Israeli material)
*C.violacea*	Tube white, limb deep violet or purple.	Scarious and serrate at the margins, glabrous. Light grey or purple.	Large conspicuous folds, yellow.	2(-3–4)
*C.tinctoria*	Tube lilac to purple on the outside, limb yellow tinged lilac to purple, throat yellow.	Scarious and serrate at the margins, glabrous. Dark gray or lilac to purple.	Very shallow, inconspicuous folds, yellow.	2
*C.tubulosa*	Light to deep yellow.	Scarious and serrate at the margins, glabrous. Light to dark grey.	Very shallow, inconspicuous folds, yellow.	2(-3–4)

Here we confirm the presence of *C.tubulosa* (Schenk) Wight ex Hook. f. 1840 and report the occurrence of three other *Cistanche* species in Israel: *C.violacea* (Desf.) Hoffmanns & Link, 1813, an entity we refer to as *C.tinctoria* (Forssk.) Beck,1904, and *C.laxiflora* Aitch. & Hemsl., 1888, and finally we describe a new species - *C.mimii* Ben-Natan & Thorogood. Our observations bridge a knowledge gap in the circumscription of the genus the Middle East and show that further work is required to resolve confusion over species limits, taxonomic diversity and distributions in the genus *Cistanche* at a global scale.

## ﻿Results

### ﻿The genus *Cistanche* in Israel

Until now, studies combining molecular analysis with careful examination of plants in the field in Israel were absent, and taxonomy in the region has been in a state of flux. In the Analytical Flora of Eretz-Israel (Eig et al. 1930), the first comprehensive account of the flora of the Palaestina region, three *Cistanche* species were reported: *C.tubulosa*, *Cistancheflava* (C.A. Mey.) Korsh. 1896, and *C.salsa* (C.A. Mey.) Beck, 1930, of which only the former is the case, as we discuss. *Cistancheflava* was described as yellow-flowered, and *C.tubulosa* was described as purple or red. However, in their second edition (Zohary and Feinbrun-Dothan 1954), both the purple and yellow-flowered forms were included under *C.tubulosa*. In the Flora Palaestina (Feinbrun-Dothan 1978), *C.tubulosa* and *C.salsa* were reported in the region, and *C.tubulosa* was described as having a ‘yellow, sometimes purple-tinged corolla’.

*Cistanchetubulosa* s.l. (Figs 2C, 5J) is a widespread S. Asian and N. African species that is common in desertic areas across the Middle East, where it is parasitic on various halophytes (e.g. *Tamarix* spp. *Atriplexhalimus* L.), often on alluvial gravel in dry riverbeds (known as ‘wadi’). It should be noted that *C.tubulosa* has been much confused with the morphologically similar *C.phelypaea*, a coastal Atlantic and western Mediterranean species that is confirmed to be genetically differentiated (Ataei et al. 2020) as well as ecologically and geographically distinct. The name *Cistanchetubulosa* s.l. has been widely used in Africa and the Middle East to South and Central Asia and China. However, as discussed by Aldughayman et al. (2024a; 2024b), this name in fact refers to a widely distributed, polyphyletic group of plants. In the phylogeny by Ataei et al. (2020), specimens identified as *C.tubulosa* (or aff. *C.tubulosa*) were placed in a ‘widespread clade’, but four specimens identified as aff. *C.tubulosa* were nested in a separate clade sister to *C.flava* along with other plants identified as *C.senegalensis* (Reut.) Beck (an entity considered by Beck-Mannagetta to be subtly distinct from *C.tubulosa*). Furthermore, plants identified as *C.tubulosa* in China correspond with the Central Asian species, *C.laxiflora* (Thorogood et al. 2021; Aldughayman et al. 2024b). The type specimen of *C.tubulosa* was lost, but recently, a specimen collected from South Sinai near the type locality was designated a neotype (Aldughayman et al. 2024a).

*Cistanchesalsa*, a species long reported to occur in Israel, is a widespread, purplish, pubescent plant that occurs from European Russia to Turkey and China in its broadest circumscription. However, like *C.tubulosa*, this name refers to a widely distributed, polyphyletic group of plants; for example, populations identified as *C.salsa* in China in fact most likely correspond with *C.deserticola* Ma. (Thorogood et al. 2021). Multiple taxa in Israel have been confused with *C.salsa* which does not occur in the region, as we discuss, and in particular the new species we describe here as *C.mimii* sp. nov., which is, in fact, closer to a further species, *C.fissa*. D. Ben-Natan and O. Hoffman first encountered *C.mimii* during a field survey in the Central Negev desert in a Nabatean loess terrace on the banks of upper Nahal Zin (Hoffman et al. 2019). The plant was conspicuous in its indumentum and had pale yellowish, strongly fragrant flowers. The plant was first identified as *Cistanchefissa* (C.A. Mey.) Beck, 1930 (Thorogood 2020, Ben-Natan D. and Fragman-Sapir O. in Flora of Israel Online (Danin and Fragman-Sapir 2016[+]) and included in a molecular phylogeny (in prep.). Close examination and molecular analysis confirm this plant is distinct, and we describe it as such below. Herbarium material identified as *C.salsa* collected from the central Negev desert and Mt. Gilead in NW Jordan should also be assigned to *C.mimii*. During further surveys between 2019 and 2024, at least nine new localities for *C.mimii* were found in the Central Negev, from the Yeruham area in the north to Mt. Sagi in the south. It is parasitic on chamaephytes in the Amaranthaceae, especially *Haloxylonscoparium* Pomel,1875.

A further species reported to occur in Israel since the account in Flora Palaestina (Feinbrun-Dothan 1978), is *C.violacea* (Desf.) Hoffmanns and Link (Figs 2A, 5G, H). This is a hairless, purple-flowered species first collected from Tunisia and reported widely from across North Africa and S. Arabian Peninsula, but again, misidentified repeatedly as *C.salsa* in Israel and Jordan. Populations in the Negev desert and the Rift Valley misidentified as *C.salsa* were determined by the authors as *C.violacea*, a species not recorded previously in Israel (cited from Israel by Ataei et al. 2020, but without referencing material examined from the region). Like *C.tubulosa*, *C.violacea* differs from *C.salsa* in being completely glabrous. Despite the superficial similarity of *C.salsa* and *C.violacea* based on their purple colouration, the presence or absence of hairs should make diagnosis unmistakable.

**Figure 1.**
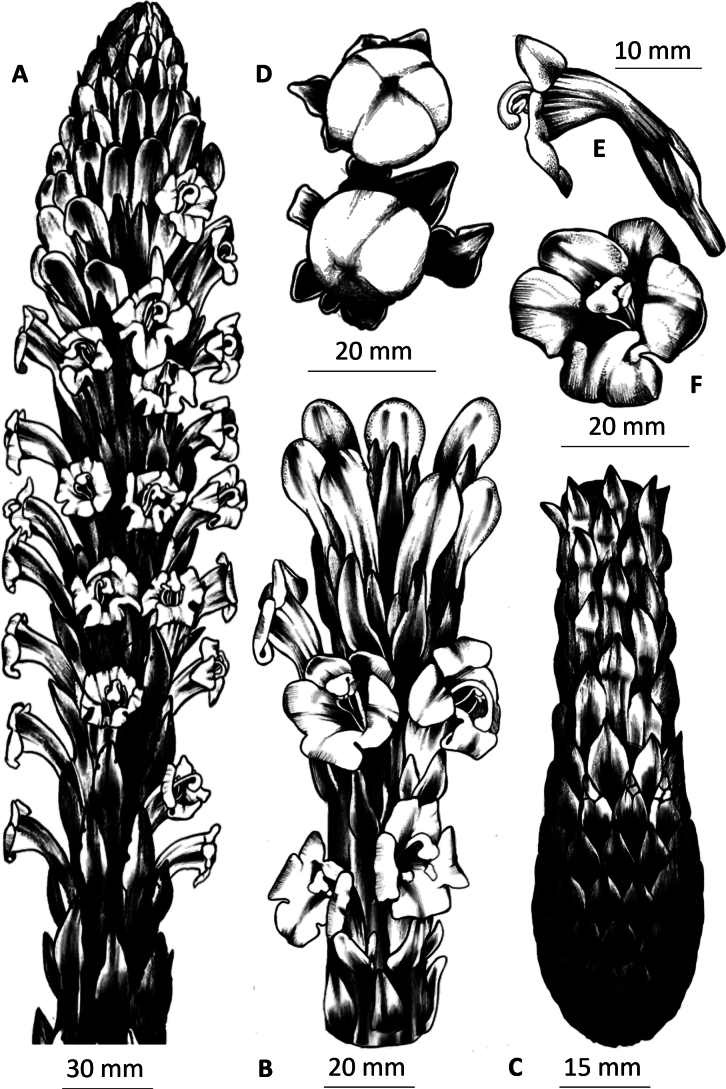
*Cistanchelaxiflora*: **A.** Habit; **B.** Inflorescence; **C.** Stem base (modified root of the plant); **D.** Fruiting capsules; **E.** Corolla in profile; **F.** Corolla showing style exserted.

Plants from the Negev desert (Figs 1, 4A–G) misidentified repeatedly as *C.salsa* and *C.tubulosa*, we assign here to *C.laxiflora*, a species not previously reported west of Iran. Finally, phylogenetic work (in prep.) reveals that a yellow-and-purple-flowered entity (Fig. 5L), which is widespread in the Middle East, is genetically distinct from the pure yellow plant, *C.tubulosa* (Fig. 5J) despite their morphological similarity. Therefore this requires recognition at the specific rank. We refer to this plant as *C.tinctoria*, for reasons we explain below.

**Figure 2.**
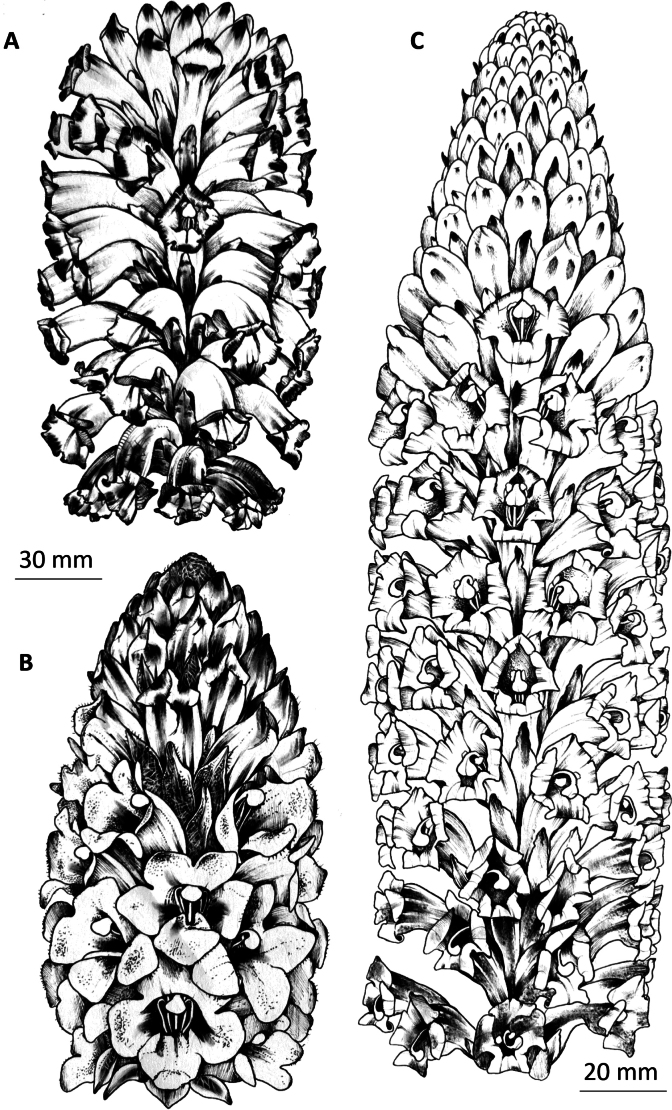
The habit of *Cistanche* species. **A.***C.violacea*; **B.***C.mimii* sp. nov; **C.***C.tubulosa*.

Here we provide descriptions for each of the five species that occur in Israel, based on examinations of both living specimens and dried material from across the region.

#### ﻿ Cistanche mimii

Taxon classificationPlantaeLamialesOrobanchaceae

Ben-Natan & Thorogood sp. nov.

429FB37D-A79E-5C8A-A821-63BE33D43E22

urn:lsid:ipni.org:names:77366410-1

##### Type materials.

***Holotype***: Israel • central Negev desert, upper Nahal Zin, SW of Avdat, loess terraces at the side of rout 40, coor 30°46'59.0"N, 34°46'06.1"E, 570 m, parasitic on *Haloxylonscoparium* Pomel, 31.Mar.2023. *Dar Ben-Natan* (HUJ135080!)

***Isotypes***: Israel • central Negev desert, upper Nahal Zin, SW of Avdat, loess terraces at the side of rout 40, coor 30°46'59.0"N, 34°46'06.1"E, 570 m, parasitic on *Haloxylonscoparium* Pomel, 31.Mar.2023. *Dar Ben-Natan* (HUJ135077!, HUJ135081!, HUJ135079!, HUJ135078!, HUJ135076!, TELA4908!)

##### Description.

A holoparasitic (achlorophyllous) herb, with a thick, glabrous stem, often lanuginose in the lower part of the inflorescence and immediately beneath, swollen and fleshy at the base. Plant short, up to 30 cm, conspicuously floccose-lanuginose to arachnoid-hairy in bud. Lower scales dense, imbricate, fleshy ovate-triangular, brown to purplish, weakly villous on margins or completely glabrous. Middle and upper scales ovate-triangular, shortly villous on the margins or completely glabrous. Floral bracts lanceolate, entire, non-scarious, non-serrulate, grey-brown to pink or dark purple, covered at least partially on outer side and on the margins with floccose-lanuginose, partly caducous hairs, often matted and capturing loess particles in between them, more rarely glabrescent and lanate only on the margins, 5–10 × 7–15 mm. Inflorescence an obtuse to broadly lanceolate raceme, up to 25 cm long and 8 cm in diameter. Floral bracts longer than calyx. Floral bracteoles 2, linear, 1–3 × 10–20 mm, grey-brown to purple, entire, non-scarious, sparsely floccose-lanuginose to glabrescent on outer side, lanate at margins, attached to the base of the calyx and usually more or less as long as the calyx. Calyx campanulate or tubular-campanulate, zygomorphic, 10–20 mm. Calyx lobes 4–5, unequal, oblong-lanceolate, entire, sometimes slightly serrulate at the margin, sparsely floccose-lanuginose to glabrescent on the exterior, lanate along the margins. The upper lobe, if present, 1–4 × 1–7, much shorter than the rest (4–5 × 6–13) or entirely missing. Corolla infundibuliform, pentamerous, almost regular, 25–35 mm. Corolla tube externally cream to flesh-coloured, sometimes tinged lilac or mauve, and internally yellow, suffused with small dark purple or black dots, with two well-pronounced yellow folds at the throat. Corolla tube arched beneath the middle. Inner tube glabrescent to slightly pubescent. Limb pale cream to fleshy pink-brown, sometimes tinged lilac, pink or mauve, especially externally (more visible in bud), 15–30 mm in diameter. Corolla lobes 5, rounded, sparsely lanuginose on the inner side, lanate at the margins, the upper ones slightly smaller than the other 3, usually wider than long. Stamens 4, inserted near the base of the tube, included. Filaments 10–17 mm, densely villous-lanate at the base, glabrous elsewhere. Anthers 2–3.5 × 4–5.5 mm, densely pilose, narrowly acuminate. Stigma lobes whitish to yellowish. Ovary elliptic, white or purplish-white with a yellow ring at base, 4–10 × 9–13 mm. Capsules globose, dark brown, 10–13 × 13–18 mm, always dehiscent with 2 valves. Fl. March-April.

##### Habitat and prevalence.

Found in Israel on loess plateaus and rocky slopes in the central Negev ridges from Yeruham to Mt. Sagi. Blooming time varies with annual rainfall, but generally falls between February to May. Collected in the 1920s by Eig, Zohary and Feinbrun-Dothan in the eastern Gilead in N Jordan as well. In the Negev it is rare, known from only 10 localities, most of which comprise small, highly local populations.

##### Hosts.

Parasitic on *Haloxylonscoparium* Pomel and possibly on other chamaephytes in the Amaranthaceae, such as *Haloxylonnegevensis* (Iljin & Zihary) Boulos 1995 and *Agathophoraalopecuroides* (Delile) Fenzl ex Bunge 1862.

##### Etymology.

The species is named in honour of Mimi (Miriam) Ron, an accomplished field botanist and surveyor and dear friend and teacher to the first author, who was a helpful companion to the field observations and collections presented in this paper.

##### Notes.

In bloom, the plants were found by the authors to emit a strong, pleasant odour, somewhat similar to that of a citron (*Citrusmedica* L. 1753.)

##### Specimens examined.

*C.mimii* (***Paratypes***): Israel • central Negev desert, Wadi Nafah near Avdat, 1.April.1945. Daniel Zohary (HUJ123605!); Israel • central Negev desert, Wadi Murra. Yoav Waisel (HUJ123608!); Israel • central Negev, South of Avdat, 17.May.1979. Prusbul L. (HUJ123667!); Israel • central Negev desert, near Avdat, Nabatean loess terrace at the banks of upper Nahal Zin, west of road 40, coor 30°46'57.2"N, 34°45'59.9"E, 560 m, Parasite on *Haloxylonscoparium* Pomel, 7.March.2018. Dar Ben-Natan (HUJ133783!); Israel • central Negev desert, south-western Makhtesh Ramon, Maavar Arod, coor 30°29'59.2"N, 34°39'22.4"E, 85 m, 5.May.2019. Yedidya Shmuel (HUJ1006363!); Israel • central Negev desert, south of Yeruham, Loess platform at the bank of upper Nahal Revivim, coor 30°57'59.6"N, 34°53'39.3"E, 490 m, Parasitic on *Haloxylonscoparium* Pomel, 12.April.2020. Dar Ben-Natan (HUJ133782!); Israel • central Negev desert, near Avdat, side of road 40, coor 30°47'01.0"N, 34°46'07.4"E, 570 m, parasitic on *Haloxylonscoparium* Pomel, 14.April.2020. Dar Ben-Natan (TELA2070!); Israel • central Negev desert, near Avdat, sides of road 40, coor 30°47'01.0"N, 34°46'07.4"E, 570 m, parasitic on *Haloxylonscoparium* Pomel, 18.April.2021. Dar Ben-Natan (HUJ133785!); Israel • Negev, rout 40, 1 km S of Avdat, sides of road. 19.Mar. 2022, Yael Orgad (HUJ133359!); Israel • Central Negev desert, SW of Yeruham, coor: 30°57'59.8"N, 34°53'39.4"E, alt: 480 m. Loess slopes above upper Nahal Revivim, at the foots of Yeruham anticline. 31.3.2023. Dar Ben-Natan (HUJ135074!, HUJ135075!); Israel • Avdat, loess, 24.4.2023, A. Singer and T. Faraj (TELA4747!); JORDAN, E. Gilead, ascent to Yabok River (Zarqa River), 8.May.1927, Eig A., Zohary M. & Feinbrun N. (HUJ123609!, HUJ123611!).

*C.fissa*: Azerbaijan • prov. Gandzha, distr. Kazach, inter pontem, Krasny-Most, et. p. Kazach-Begly, 2.May.1927. A. Kolakowsky (HUJ133396!).

*C.salsa*: Turkey • Env. of Chihanbeyli, 25.May.1953. B. Kasapligil (HUJ123619!).

#### ﻿ Cistanche tubulosa

Taxon classificationPlantaeLamialesOrobanchaceae

(Schenk) Wight ex Hook. f.

C3EA47B1-4180-584B-A962-CC69FC406843

≡ Phelypaeatubulosa Schenk, Sp. Pl. Aegypt. 23–24 (October 1840) [basionym].

##### Description.

A holoparasitic (achlorophyllous), glabrous herb, with a stout stem, swollen at the base. Plant robust, 30–130 cm, stem dirty-white or grey, 15–40 mm in diameter. Lower scales dense, constricted, entire, triangular, yellowish-white, grey or dark grey, 5–10 × 5–10 mm. Middle and upper scales and floral bracts triangular-lanceolate, entire, scarious and variably, often markedly serrate along the margins, dirty-white to grey, 7–15 × 15–30 mm. Inflorescence a dense, many-flowered spike, 20–100 cm long, 4–15 cm in diameter. Floral bracts about as long as calyx or somewhat longer. Floral bracteoles 2, linear or lanceolate, obtuse, 2–6 × 10–20 mm, white to grey, scarious and serrate along the margin, attached to the calyx base and equal or slightly shorter than calyx. Calyx tubular-campanulate, dirty-white to grey, 10–22 mm. Calyx lobes 5, equal or subequal with one slightly shorter lobe, oblong-rounded, entire, scarious and serrate at the margin. The upper lobe shorter than the rest by up to 3 mm. Corolla infundibuliform, pentamerous, 35–45 mm. Corolla tube bright yellow, with two shallow, yellow folds inside. Corolla tube arched beneath the middle. Inner tube more or less short-pubescent around the filament bases and glabrous elsewhere. Limb usually bright yellow, sometimes slightly tinged crimson (especially conspicuous in bud), 15–30 mm in diameter. Corolla lobes 5, rounded, equal or the upper ones slightly smaller than the other 3, usually wider than long. Stamens 4, inserted at the base of the tube, included. Filaments 20–25 mm, densely pubescent at the base and weakly, very short-pubescent or glabrous above. Anthers 3 × 5 mm, densely lanuginose, rounded-elliptic to apiculate. Ovary ovate, 4–5 × 10–12 mm, white or yellowish-white with a yellow ring at base, dark brown when dry. Capsules ovoid-globose, 12–20 × 15–20 mm, dehiscent with 2(-3–4) valves. Fl. January-April.

##### Habitat and prevalence.

Wadis in the desert. Along the Rift Valley from the southern Jordan River to Eilat, in the eastern and southern Judean desert, and the central and southern Negev desert. Fairly common in its habitat.

##### Host.

Mostly *Tamarix* ssp. from *T.nilotica* (Erenb.) Bunge. group and *Atriplexhalimus* L. Probably other desert shrubs, especially in the Amaranthaceae.

##### Notes.

see *C.tinctoria*. *C.tubulosa* can be found in mixed populations with either *C.laxiflora* or *C.violacea*, and there is evidence of hybridization with the latter.

##### Specimens examined.

Israel • (Palestine), Lower Jordan Valley, plain of Jerico, 27.May.1905. Dinsmore J. E. (HUJ123660!); Israel • Judean desert, Wadi ‘Ghar, 26.March.1929. Gabrielith R. (HUJ123662!); Israel • southern Negev, Wadi ‘Ideid (Nahal ‘Arod), between Khiar ‘Ideid (Be’erot ‘Oded) and Gebel ‘Oreif (Har ‘Arif), 21.April.1946. Zohary D. (HUJ123669!); Israel • Arava Valley, Ein Hosb (En-Hatzeva), 9.April.1950. Near spring, on roots of *Tamarixmaris-mortui* Gutmann, Zohary D. (HUJ123664!); Israel • Arava Valley, 82 km N of Eilat, Wadi bed, 13.March.1951. Orshan G. & Zohary D. (HUJ123666!); Israel • Env. of Dead Sea, env. of Masada, 7.March.1954. D’Angelis Y. (HUJ123626!); Israel • Env. of south Dead Sea, 23.March.1954. Zohary M. (HUJ123627!) Israel • Dead Sea area, En-Gedi, Nahal David, 18.March.1961. Fuchs S. (HUJ123625!); Israel • central Negev, Ein Avdat, 8.April.1968. Prusbul L. (HUJ123668!); Israel • (Palestine), 15 km S of Jericho, on edge of Dead Sea, 13.February.1987. Musselman L. Y. (HUJ123623!, HUJ123624!); Israel • (Palestine), Samaria, Far’a Valley, Nahal Tirtsa, SE of Adam junction, 26.February.2001. Ur Y. (HUJ123630!); Israel • Negev desert, Makhtesh Ramon, Ein-Saharonim, coor. 30°36'00.6"N, 34°56'17.0"E, 300 m, Wet, somewhat saline sandy banks of a small freshwater stream, 14 April 2020. Dar Ben-Natan (HUJ1005812!). Israel • N. Arava Valley, Lower Ein Gidron, N. of Hatzeva, sand and grit, near flowing water, coor. 30°47'45.7"N, 35°16'34.3"E, 190 m, 25.May.2022. parasitic on *T.nilotica* (Ehrenb.) Bunge. Dar Ben-Natan (HUJ1000047!); Israel • Arava Valley, north of Tzofar, western side of road 90, Nahal Shivya, coor 30°34'00.5"N, 35°11'16.3"E, 50 m, parasitic on *Atriplexhalimus* L., containing fruiting capsules dehiscent by 3 and 4 valves 7.May.2021. Dar Ben-Natan (TELA2796!); Israel • Judean Desert, Side of Arad to Dead Sea road, coor 31°10'39.5"N, 35°17'47.5"E, 150 m, parasitic on *Atriplexhalimus* L., containing fruiting capsules dehiscent by 4 valves 7.May.2021. Dar Ben-Natan (TELA2797!); Israel • Judean Desert, Side of Arad to Dead Sea road, Nahal Zahav, coor 31°10'23.3"N, 35°18'40.7"E, 110 m, parasitic on *Atriplexhalimus* L., containing fruiting capsules dehiscent by 4 valves 7.May.2021. Dar Ben-Natan (HUJ134826!); Israel • Judean desert, Lower Nahal Og, containing capsules dehiscent by 3–4 valves, 1.Oct.2022, Dar Ben-Natan (HUJ1005811!);

#### ﻿ Cistanche tinctoria

Taxon classificationPlantaeLamialesOrobanchaceae

(Forssk.) Beck

635D3BBA-B605-5AD4-A15B-A8A9D5BB33DE

##### Description.

A holoparasitic (achlorophyllous), glabrous herb, with a stout stem, swollen at the base. Plant robust, 30–150 cm, stem purple to dark purplish-brown, 15–40 mm in diameter. Lower scales dense, constricted, entire, triangular, dark, 5–10 × 5–10 mm. Middle and upper scales and floral bracts triangular-lanceolate, entire, scarious and markedly serrate along the margins, purple to dark purplish-brown, 7–15 × 15–30 mm. Inflorescence a dense, many-flowered spike, 20–150 cm long, 5–15 cm in diameter. Floral bracts about as long as calyx or somewhat longer. Floral bracteoles 2, linear or lanceolate, obtuse, 2–6 × 10–20 mm, pale lilac to dark purple, scarious and serrate along the margin, attached to the calyx base and equal or slightly shorter than calyx. Calyx tubular-campanulate, pale lilac to dark purple, 10–22 mm. Calyx lobes 5, equal or subequal with one slightly shorter lobe, oblong-rounded, entire, scarious and serrate at the margin. The upper lobe is shorter than the rest by up to 3 mm. Corolla infundibuliform, pentamerous, 35–45 mm. Corolla tube tinged lilac to purple externally (conspicuous, especially in bud), yellow within with two shallow yellow folds inside. Corolla tube arched beneath the middle. Inner tube more or less short-pubescent around the filament bases and glabrous elsewhere. Limb tinged pale violet to deep purple (mostly on upper lobes, most noticeable in bud), 15–30 mm in diameter. Corolla lobes 5, rounded, equal or the upper ones slightly smaller than the other 3, usually slightly wider than long. Stamens 4, inserted at the base of the tube, included. Filaments 20–25 mm, densely pubescent at the base and weakly, very short-pubescent or glabrous above. Anthers 3 × 5 mm, densely lanuginose, rounded-elliptic to apiculate. Stigma lobes white to pale yellow. Ovary ovate, 4–5 × 10–12 mm, white or yellowish-white with a yellow ring at base, dark brown when dry. Capsules ovoid-globose, 12–20 × 15–20 mm, dehiscent with 2 valves. Fl. January-April.

##### Habitat and prevalence.

Wadis in the arid desert, on sandy or loessial soils. Also in planted groves of *Tamarixaphylla* (L.) Karsten. at the margins of agricultural plots. In the region, it is restricted to the southern Arava Valley, from Yotvata to Eilat. It seems this species occurs in the region at the margins of its distribution, and is very rare in Israel – currently known from only 15 localities. Is it present in S. Sinai, and probably also in Jordan on the eastern side of the Arava Valley.

##### Host.

Observed in the region on *Tamarixaphylla* (L.) Karsten. Other potential hosts include: *Haloxylonpersicum* Bunge, *Caroxylonimbricatum* (Forssk.) Moq. And *Caroxylongaetulum* (Maire) Akhani & Roalson. Probably other desert shrubs, especially in the Amaranthaceae.

##### Notes.

We initially considered these populations to be a colour morph of *C.tubulosa*, until phylogenetic work confirmed these species are genetically distinct (in prep.). No clear, discernible morphological differences were observed between the two species, beside the purple coloration of *C.tinctoria*. Further work may confirm ecological differences such as habitat and host range; for example *C.tubulosa* (as circumscribed here) is not reported to parasitize any the host species described for *C.tinctoria*, and is absent from sandy substrates in the region. The taxa are not known to co-occur in the region.

##### Specimens examined.

Israel • Arava Valley, Samar. Sands, 4.3.1983, A. Liston (HUJ123613!); Israel • S. Arava Valley, sand, crop fields north of Yotvata, coor 29°54'22.2"N, 35°04'37.9"E, 80 m, parasite on *Tamarixaphylla* (L.) Karsten, purple form, 17.January.2013. Dar Ben-Natan (TELA2073!); Sinai • Tiran Island, S. peninsula, coastal foothills N. of Gebel Tiran, small sandy wadis between low sandstone questas, 13.December.1968. N. Tadmor & A. Danin (HUJ123614!).

#### ﻿ Cistanche violacea

Taxon classificationPlantaeLamialesOrobanchaceae

(Desf.) Hoffmanns & Link

33A9E317-0E80-519D-87FD-103F39C457A7

≡ Phelypaeaviolacea Desf., Fl. Atlant. 2: 60, t. 146 (1798) [basionym].
≡ Orobancheviolacea (Desf.) Wallr., Orob. Gen. 70 (1825).
≡ Cistancheviolacea (Desf.) Beck, Biblioth. Bot. 19: 267 (1890), comb. superfl.

##### Description.

A holoparasitic (achlorophyllous), glabrous herb. Plant robust, 10–80 cm, stem white to grey or purple, 10–20 mm in diameter, swollen at the base. Lower scales dense, constricted, entire, triangular, yellowish-white to dark grey, 5–10 × 5–10 mm. Middle and upper scales and floral bracts triangular-lanceolate, entire, scarious and strongly serrate along the margins, white or lilac to purple or dark purplish-brown, 7–15 × 15–30 mm. Inflorescence a dense spike, up to 30 cm long, 5–15 cm in diameter. Floral bracts about as long as calyx or somewhat longer. Floral bracteoles 2, linear or lanceolate, obtuse, 2–6 × 10–22 mm, white, grey or purple, scarious and serrate along the margins, attached to the calyx base and equal or slightly shorter than calyx. Calyx tubular-campanulate, white, lilac, purple or purplish-brown, 10–22 mm. Calyx lobes 5, subequal, oblong-rounded, entire, scarious and serrate along the margins. The upper lobe is shorter than the rest by up to 3 mm. Corolla infundibuliform, pentamerous, 35–50 mm. Corolla tube white, with two well-pronounced, conspicuous yellow folds inside. Corolla tube arched beneath the middle. Inner tube more or less pubescent around the filament bases and glabrous elsewhere. Limb pale to deep purple-violet, 15–25 mm in diameter. Corolla lobes 5, rounded, equal or the upper ones slightly smaller than the other 3, usually wider than long. Stamens 4, inserted at the base of the tube, included. Filaments 18–21 mm, densely pubescent at the base and very sparsely, very short-pubescent or glabrous above. Anthers 2–3 × 4.5–5.5 mm, densely pilose, acuminate. Ovary ovate, 5–10 × 10–15 mm, white with a yellow ring at base. Stigma lobes whitish or yellowish. Capsules ovoid-globose, 12–20 × 15–20 mm, dark brown when dry, dehiscent with 2(-3–4) valves. Fl. February-April.

##### Habitat and prevalence.

*C.violacea* occurs on alluvial gravel and clay substrates (mostly loess and marl) in wadies along the Rift Valley from the Dead Sea to Eilat, in the eastern and southern Judean desert and rarely in the central Negev, and on stabilized or shallow sands over chalk in the western Negev desert. It is fairly common, though much less than *C.tubulosa*, occurring often in mixed populations with the latter along the Rift Valley. Present also in SE Jordan (Dead Sea area, Arava Valley, Wadi Rum), and occasionally mistaken for *C.salsa* there as well.

##### Host.

Mostly *Anabasisarticulata* (Forssk.) Moq. and *Atriplexhalimus* L., more rarely observed on *Haloxylonnegevensis* (Iljin & Zohary) L.Boulos. Other possible hosts include *Tamarixnilotica* (Erenb.) Bunge. and *Anabasissetifera* Moq. 1840.

##### Notes.

Aside from coloration, *C.violacea* can be difficult to distinguish from *C.tubulosa* and *C.tinctoria*, which makes determination of dry herbarium specimen problematic. Moreover, in some mixed populations in the Rift Valley, morphological intermediates occur which are most likely hybrids of *C.violacea* and *C.tubulosa* (Fig. 5I), based on molecular work (in prep.). In the western Negev desert, however, populations appear to be distinct. Besides pigmentation and stature, the very well-pronounced yellow folds in the throat of the corolla and anther morphology proved to be important and useful diagnostic characteristics for *C.violacea* – especially in dry specimens, both in the field and the herbarium.

##### Specimens examined.

Israel • Judean desert, Massada, 7.April.1942. Zohary D. (HUJ123604!); Israel • Judean Desert, steppes, 4.1942, Issac Halevv (HUJ123663!); Israel • Dead sea area, Wadi Fuqra (Nahal Zin), 18.March.1967. Avishai M. (HUJ123601!, HUJ123602!); Israel • Judean desert, east of Arad, small wadi, 11.April.1967. Kollman F. (HUJ123600!); Israel • Judean desert, btw. Mezad Zafit and Mezad Tamar, hard rock, 11.March.1972. Zohary D. (HUJ123603!); Israel • Western Negev, 10 km NNW from Nizzana, Nahal Lavan, sandy loess, 25.March.1986. Danin A. (HUJ123612!); Israel • Hatzeva field school, 13.March.1997. Pazy B. and Plitmann U. (HUJ123606!); Israel • Judean Desert, Nahal Metsada west of Metsada, 60 m, 21.February.2012. Dar Ben-Natan (TELA2076!); Israel • West Negev, near Shivta, on route 211. Mar.2015. (HUJ133888!); Israel • Judean Desert, Arad to Dead Sea road, 280 m, parasite on *Atriplexhalimus* L., together with *C.tubulosa* (Schenk) Wight ex Hook. f. and possible intermediate forms, 3.March.2015. Dar Ben-Natan (TELA2077!) Israel • northern Arava Valley, Idan reservoir, 180 m, together with *C.tubulosa* (Schenk) Wight ex Hook. f. and possible intermediate forms between the two species, 19.March.2019. Dar Ben-Natan (TELA2072!); Israel • western Negev desert, Nizzana, Han Nizzana yard, shallow sand over loam, coor 30°53'06.9"N, 34°25'29.3"E, 190 m, parasite on *Anabasisarticulata* (Forssk.) Moq. 12.March.2020. Dar Ben-Natan (HUJ133784!); Israel • Dead Sea area, east of road 90, across from massada, white clay, together with *C.tubulosa* (Schenk) Wight ex Hook. f., coor: 31°19'23.5"N, 35°23'11.3"E, -380 m, some possibly parasitic on *Anabasissetifera* Moq., some on *Atriplexhalimus* L., containing fruiting capsules dehiscent by 3 and 4 valves, 7.May.2021. Dar Ben-Natan (TELA2799!); Israel • Dead Sea area, estuary of Nahal Rahaf, western side of road 90, coor 31°17'14.9"N, 35°22'53.8"E, -380 m, possibly parasitic on *Atriplexhalimus* L., containing fruiting capsules dehiscent by 4 valves 7.May.2021. Dar Ben-Natan (TELA27800!); JORDAN, 40 km S of Ma’an. “Nubian sand” fields. 18.Apr.1929. A. Eig and M. Zohary (HUJ123672!); Sinai • Gebel el Tih, 110 km S. of Nakhl, Badlands of Ghareb formation at the foot of Igma Plateau, 22.November.1969. N. Tadmor (HUJ123615!); Sinai • w. foothills of Gebel Ya’allaq, wadi bed and terraces with variable sand cover of 0–50 cm, 20–30.March.1973. Corolla violet-white (not yellow), I. Noy-Meir (HUJ123617!); Sinai • N. Sinai, Umm Qataf, 2 km north of Nizzana, sands covering limestone hill, 9.April.1976. Avinoam Danin (HUJ123616!).

#### ﻿ Cistanche laxiflora

Taxon classificationPlantaeLamialesOrobanchaceae

Aitch. & Hemsl.

91CF62F4-D33C-500E-B789-F26C0228E00A

##### Description.

A holoparasitic (achlorophyllous), glabrous herb, with a stout stem, swollen at the base. Plant robust, 30–120 cm, with white or pale grey to pale lilac stem, 20–40 mm in diameter. Lower scales dense, constricted, entire, wide-triangular, yellowish, 10 × 10 mm. Middle and upper scales and floral bracts triangular-lanceolate, fleshy, entire, non-scarious and non-serrate, or sometimes very slightly serrulate, white or grey to pale lilac, 10 × 25 mm. Inflorescence a more or less lax spike with 40–200 flowers, 30–100 cm long, 5–8 cm in diameter, with or without a stem up to 20 cm below. Flowers at the base of the inflorescence sometimes with pedicels up to 15 mm long. Floral bracts about as long as calyx, or somewhat longer. Floral bracteoles 2, linear, obtuse, 2–4 × 10–15 mm, white, pale grey or pale lilac, fleshy, sometimes slightly serrulate at the margin, entire, non-scarious, attached to the calyx base and slightly shorter than calyx. Calyx tubular-campanulate, 13–17 mm. Calyx lobes 5, equal to subequal, oblong-rounded, entire, slightly scarious along the margins. The apical lobe 3–5 × 8–12, usually slightly shorter than the rest (3–5 × 11–15), sometimes the two bottom lobes slightly shorter than the side lobes (by 1 mm at most). Corolla infundibuliform, pentamerous, 30–35 mm. Corolla tube white, sometimes tinged lilac externally, with two yellow, flat, inconspicuous folds inside. Corolla tube arched at the middle or just beneath. Tube 15–16 mm below the arch, and 15–20 mm above. Inner tube more or less very short-pubescent around the filament bases, glabrous elsewhere. Limb whitish to very pale mauve or violet, 20–25 mm in diameter. Corolla lobes 5, rounded, equal or the upper ones slightly smaller than the other 3, usually wider than long. Stamens 4, inserted at about 1/3 the total corolla length, included. Filaments 15–20 mm, more or less short-pubescent at the base, glabrous or very sparsely, very short-glandular elsewhere. Anthers 3–4 × 2 mm, sparsely lanuginose, apically rounded, usually with a small tubercule at the base. Stigma lobes white. Ovary tubular-elliptic, white or yellowish-white with a yellow ring at base, dark brown when dry, 10–15 × 5–8 mm. Capsules ovoid-globose, 13–25 × 5–15 mm, dehiscent with 2–4 valves. Fl. March-April.

##### Habitat and prevalence.

Sandy-loessial soils and alluvial gravel near springs or over high-level ground water in the desert. This species is extremely rare in the region, currently known from 4 localities and threatened by depleting levels of ground water.

##### Host.

Probably *Tamarixnilotica* (Erenb.) Bunge, 1852 (Tamaricaceae), which we observed in association with the parasite in all four localities.

##### Notes.

All four populations co-occur with *C.tubulosa*. The two distinct species co-occur in sympatry and although they bloom simultaneously, intermediate morphotypes have not been observed – although molecular data shows there are rare events of hybridization (Ein-Gidron).

##### Specimens examined.

Israel • Arava Valley, Ein Hosb (En-Hatzeva), 9.April.1950. Near spring, on roots of *Tamarixmaris-mortui* Gutmann, Zohary D. (HUJ123665!); Israel • Negev desert, Makhtesh Ramon, Ein-Saharonim, coor. 30°36'06"N, 34°56'17.2"E, 300 m, Wet, somewhat saline sandy banks of a small freshwater stream, 14 April 2020. Dar Ben-Natan and Mimi Ron (HUJ133363!, HUJI133366!, HUJ133364!, HUJ133361!, HUJ133365!, HUJ133369!, TELA2050!, TELA2051!); Israel • N. Arava Valley, Lower Ein Gidron, N. of Hatzeva, sand and grit, near flowing water, coor. 30°47'46.9"N, 35°16'34.4"E, 190 m, 22.April.2021. parasitic on *T.nilotica* (Ehrenb.) Bunge? together with *Imperatacylindrica* (L.) Raeusch. Dar Ben-Natan and Mimi Ron (HUJ133398!); Israel • N. Arava Valley, Lower Ein Gidron. Dry capsules with 4 valves. 6.Oct.2021. Dar Ben-Natan and Mimi Ron (HUJ134741!); Israel • E Negev, Ein-Akrabim, coor: 30°53'34.8"N, 35°09'13.9"E. Parasitic on *T.nilotica (Ehrenb.) Bunge*. 26.Mar.2022, Dar Ben-Natan and Mimi Ron (HUJ134766!, HUJ134769!); Israel • Makhtesh Ramon, Ein Saharonim, 30.3.2023, Dar Ben-Natan (TELA!); Israel • Arava valley, lower Ein-Gidron, coor: 30°47'46.4"N, 35°16'34.3"E, alt: 180 m. Hidden in the undergrowth at the S. bank of the wadi, near the eastern edge of the water stream, on wet, saline silt-grit. Probably parasitic on *T.nilotica* (Ehrenb.) Bunge. 14.Apr.2024 Dar Ben-Natan and Mimi Ron (HUJ!1006364!); Israel • Arava valley, lower Ein-Gidron, coor: 30°47'46.5"N, 35°16'33.8"E, alt: 180 m. Hidden in the undergrowth at the S. bank of the wadi, near the eastern edge of the water stream, on wet, saline silt-grit. Probably parasitic on *T.nilotica* (Ehrenb.) Bunge. 14.Apr.2024. Dar Ben-Natan and Mimi Ron (HUJ1006365!, HUJ1006366!, HUJ1006367!, HUJ1006368!, HUJ1006369!).

## ﻿Discussion

Despite falling within a regional centre of diversity of the genus *Cistanche*, Israel and adjacent territories have been neglected from prior work. Records of the species occurring in Israel are confused and understate diversity in the region. Moreover, we present evidence of phenotypic plasticity, previously overlooked, that may extend beyond the region covered by our assessment. This is of concern because careful examination of populations and reliable identification at a regional level is required to inform broader-scale treatments based on DNA sequence data, and conservation practice.

Importantly we observed fruiting capsules dehiscent by 3 or 4 valves in multiple taxa (*C.laxiflora*, and occasionally in *C.tubulosa* and *C.violacea*) across their distribution in the region. In each case, variation in valve number corresponded with an increase in placentae (i.e. 4 in 2-valved capsules, and 5–8 in 3–4-valved capsules). Capsules with more than two valves have not been recorded in any species belonging to the recently delineated Widespread Clade; indeed, the only other species of *Cistanche* reported to produce fruiting capsules with 3 valves are the poorly-known species *C.ridgewayana* and *C.trivalvis* (Trautv.) Korsh. 1896, both of which are established to belong to a separate, Southeast-Asian clade, characterized by hirsute floral parts (Ataei et al. 2020). From our data, we conclude that valve number is unreliable as a defining characteristic, at least in the Widespread Clade and possibly genus-wide (we have not observed 3–4-valved capsules in *C.mimii*). Our assessment indicates that circumscription and variability are insufficiently understood, and that further work is required to resolve the relationships in this complex and problematic genus in the Middle East.

### ﻿Taxonomic considerations

#### ﻿(i) *Cistanchetubulosa* and *C.tinctoria*

Recent molecular work identified the presence of two morphologically similar but phylogenetically distinct entities that co-occur in Israel and the wider Middle East. Monomorphic populations of *C.tubulosa* in the Negev and the Judean Desert possess a uniformly bright yellow corolla and whitish to grey calyx, bracts and bracteoles. In the southern Arava Valley, specimens show variable degrees of pigmentation including dark-purple calyx, bracts and bracteoles, and corolla tube and limb tinged purple; these are now established to belong to the genetically distinct, co-occurring *C.tinctoria* (Forssk.) Beck. Presumably because of their purple pigmentation, these populations have also been occasionally misidentified as *C.salsa* (C.A. Mey.) Beck, 1930, which has hairy bracts (see below). The most reliable diagnostic for distinguishing *C.tubulosa* and *C.tinctoria* is the absence of purple pigmentation in the former; *C.tinctoria* is usually strongly purple-tinted. The name *C.tubulosa* was synonymised under *C.tinctoria* until a neotype was assigned recently using material from near the original type location in Sinai (Aldughayman et al. 2024a). This neotypification was justified on the basis that *C.tubulosa* was still the most widely applied name (used in at least 21 regional floras). The basionym *Orobanchetinctoria* was published in 1775 by Peter Forsskål for a plant he collected in Yemen (Forsskål 1775), and the date of publication of this basionym predates that of *C.tubulosa*. Therefore *C.tinctoria* is a sensible name to apply to yellow and yellow-purple plants that are now known to be genetically distinct from *C.tubulosa*. A comparison of diagnostic characters for these species is given in Table 1.

#### ﻿(i) *Cistanchemimii* sp. nov.

**Figure 3.**
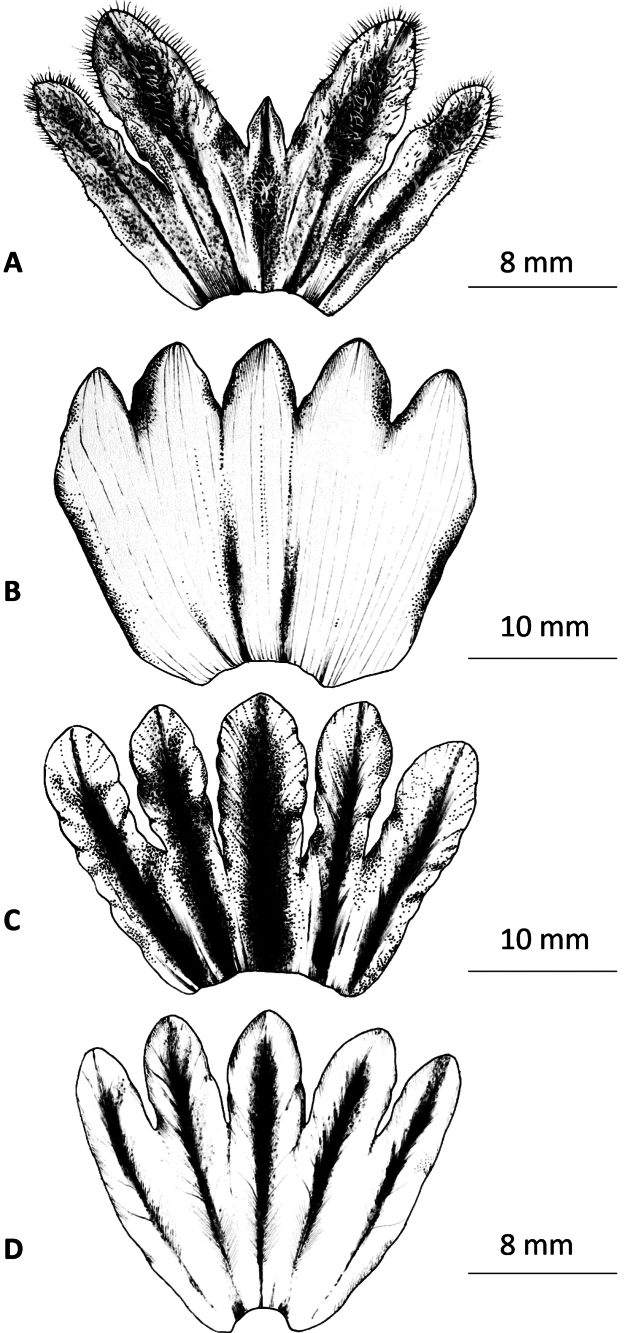
The calyx morphology of *Cistanche* species. **A.***C.mimii* sp. nov; **B.***C.tubulosa*; **C.***C.violacea*; **D.***C.laxiflora*. We could discern no difference between the calyx morphology of *C.tubulosa* and *C.tinctoria*, other than the presence of purple pigmentation in the latter.

**Figure 4.**
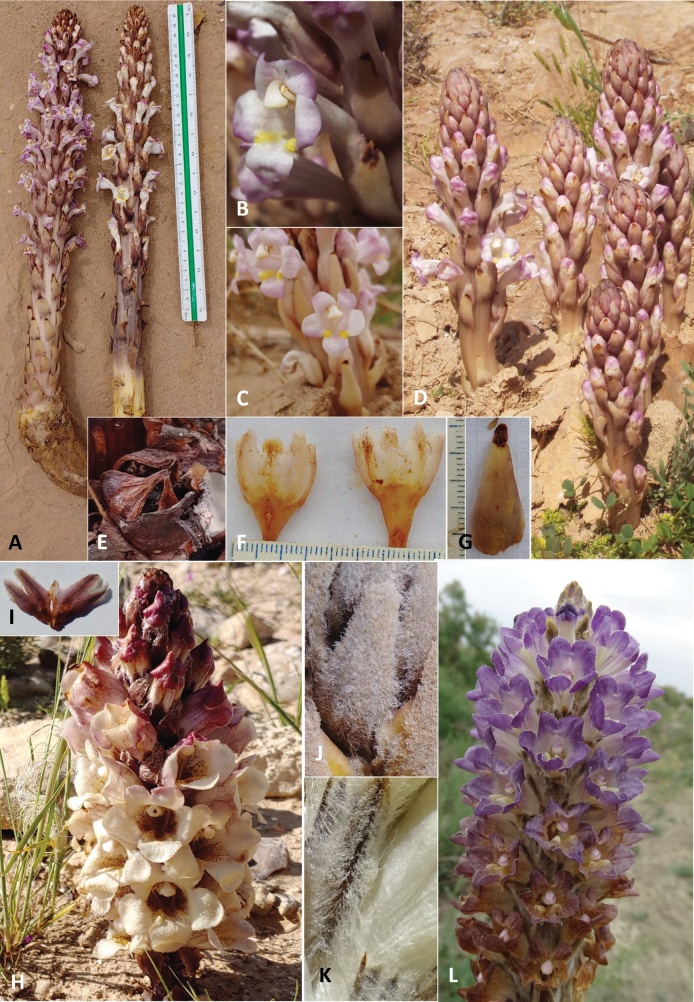
*Cistanchelaxiflora* (Ein Saharonim): **A.** Whole plant; **B.** Flower, photo by Yoav Ramon; **C.** Flowers; **D.** Habit of plant growing in the Ein Saharonim spring in Makhtesh Ramon, photo by Yoav Ramon; **E.** 4-valved dehiscent capsule; **F.** Dissected calyces; **G.** Bract; *Cistanchemimii* sp. nov.; **H.***C.mimii* sp. nov. S. of Yeruham, occurring at the northern limit of the known range in Israel; **I.***C.mimii* sp. nov. dissected calyx (specimen from S. of Yeruham); **J.** Arachnoid indumentum of juvenile specimen (specimen from near Avdat). **K.** Villous-arachnoid indumentum of *C.fissa* (specimen from Azerbaijan); **L.***C.fissa* in its type locality in Azerbaijan. Photos K-L by Renata Piwowarczyk.

Here we show that *C.mimii* is a distinct taxon that requires recognition at the specific rank. The closest known species is *C.fissa*, which itself, is a confused taxon for which there are conflicting accounts in the literature. For example, despite hairiness being a key diagnostic feature for *Cistanche*, the stem of *C.fissa* is described as pubescent in the last monograph of the genus (Beck-Mannagetta, 1930) but glabrous by other authors (Ataei 2017). The type specimen of *C.fissa* seems to have a sub-glabrous stem. Material from Azerbaijan examined by us has a sub-villous stem, meanwhile *C.mimii* has a stem that is subglabrous to sparsely lanuginose only immediately beneath the inflorescence, and glabrous elsewhere. Israeli material has a conspicuous floccose-lanuginose indumentum in bud (Fig. 4J) and conspicuous white-woolly indumentum on the margins and inner surface of the corolla lobes, a zygomorphic calyx with a diminutive or absent apical lobe (Fig. 3A), and almost regular corolla (Figs 2B, 4H) – all of which are consistent with *C.fissa*, which molecular work shows is closely related, although distinct (in prep.). However, the new species is somewhat distinct from material previously reported in that the corolla is yellowish to cream-tinted (not pure white) flushed mauve or lilac (rather than blue-purple – see *C.fissa*, Fig. 4L) and more or less pubescent, with conspicuous small dark dots inside the tube. The indumentum on the floral parts of *C.mimii* is mostly pubescent-lanuginose, often quite thick, matted and capturing wind-blown loess particles, and partially caducous (vs. finer villous-arachnoid, non-caducous indumentum in *C.fissa* – see Fig. 4K). Additionally, the flowers emit a fragrant, citrus-like scent, unreported for *C.fissa* in literature, or our correspondence with botanists who have examined living material of that species. All these traits, together with the disjunct distribution and the different habitat and hosts, show that the Israeli populations requires recognition at the specific rank.

**Figure 5.**
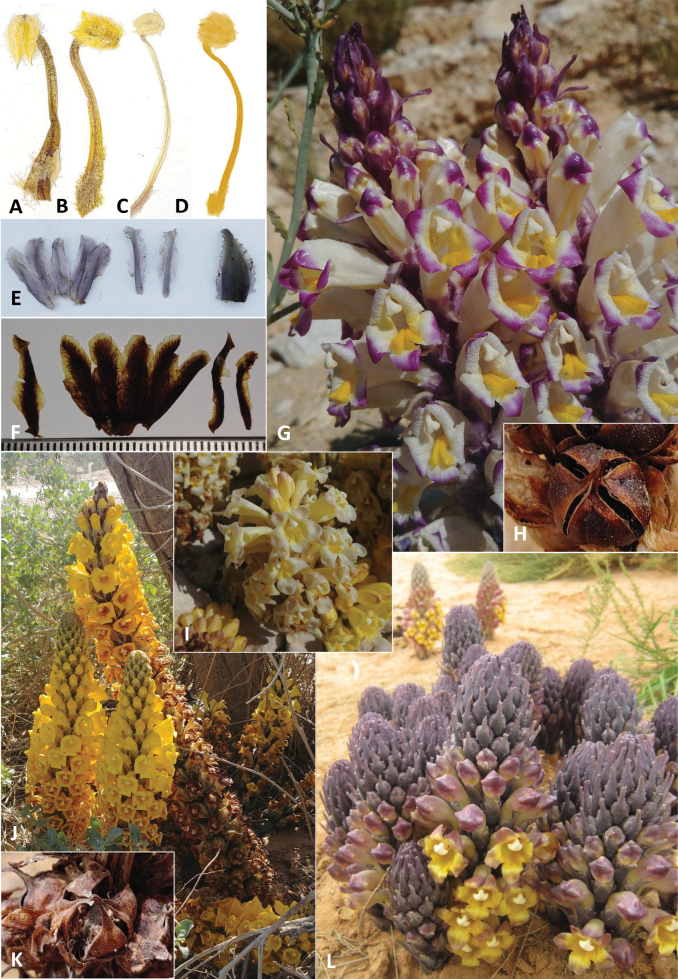
**A–D.** Stamens of *C.mimii* sp. nov., *C.violacea*, *C.laxiflora*, and *C.tubulosa*; **E.***C.violacea*, Left to right - dissected calyx, bracteoles and bract (specimen from Nizzana); **F.***C.tubulosa*, Left to right – bract, dissected calyx and bracteoles (specimen from Ein Saharonim); **G.***C.violacea* near Ein Yahav (N. Arava Valley); **H.** 4-valved dehiscent capsule of *C.violacea* (specimen from Nahal Mor, Dead Sea area); **I.** Putative Hybrid between *C.tubulosa* and *C.violacea* near Ein Yahav (N. Arava Valley); **J.** Habit of *Cistanchetubulosa*, parasitic on *Atriplexhalimus* near Tzofar (Arava Valley); **K.** 4-valved dehiscent capsule of *C.tubulosa* near Arad to Dead Sea road (Judean Desert); **L.** Habit *C.tinctoria* in Yotvata’s agricultural fields, parasitic on *Tamarixaphylla*.

*C.mimii* has been confused with *C.salsa*. The latter species, reported widely from Turkey, east to China, is also included in the Flora Palaestina (“C. Negev; Dead Sea area; E. Gilead” – 3:209-210), probably based on just three collections in the HUJI herbarium – ISRAEL, central Negev desert, Wadi Nafah near Avdat, 1.April.1945. Daniel Zohary (HUJ123605!); ISRAEL, central Negev desert, Wadi Murra. Yoav Waisel (HUJ123608!); JORDAN, E. Gilead, ascent to Yabok River (Zarqa River), 8.May.1927, Eig A., Zohary M. & Feinbrun N. (HUJ123609!, HUJ123611!). We consider all of these to belong to *C.mimii* (Figs 2B, 4H–J). Records of *C.salsa* from the Dead Sea area are taken from Post (1933) and refer to the eastern bank of the Dead Sea, which is in Jordan territory (and is probably also a case of mistaken identification).

*Cistanchesalsa* and *C.fissa* are closely related according to DNA sequence data from putative *C.fissa* sourced from Afghanistan and Azerbaijan, and *C.salsa* from southwestern Asia and China (Ataei 2017). These taxa are characterized by the woolly indumentum of the floral parts that is common among the species of the (predominantly) Southwest-Asian clade (Ataei et al. 2020). However, *C.mimii* and *C.fissa*, are both distinguished from the former by the deeply dissected calyx (Fig. 3A) with a reduced or absent apical calyx lobe, and hairy corolla limb; hence their assignment to different sections (now refuted) in the only monograph of the genus (Beck-Mannagetta 1930). We consider the occurrence of *C.salsa* in Israel and western Jordan to be doubtful. Recent molecular work did not include material of *C.fissa* but showed that *C.mimii* and *C.salsa* are distinct. *C.salsa* s.l. is not known currently from Israel or western Jordan, but may be present in eastern Jordan. A comparison of diagnostic characters for these species is given in Table 2.

**Table 2.** Diagnostic characters for species with hairy calyces in the region (*C.mimii* sp. nov., *C.fissa* and *C.salsa*). Details for *C.fissa* are based on examination of photographs of the holotype and of living specimens from the type locality, and for *C.mimii* on living and dried material from Israel (see *Specimens Examined*). Details for *C.salsa* are adapted from the monograph (Beck-Mannagetta 1930), other treatments (e.g. Ataei et al. 2020) and our examination of photographs of the holotype.

Species	Calyx lobes	Corolla colour and indumentum	Inflorescence stem indumentum
*C.salsa*	Equal or subequal. Indumentum white-woolly in bud.	Pale lilac or white, with two yellow folds at the throat. Limb glabrous or sparsely ciliate along the margins.	Lanuginose.
*C.mimii* sp. nov.	Unequal. Apical calyx lobe considerably shorter than the others or missing entirely. Indumentum floccose-lanuginose in bud.	Pale cream, sometimes limb tinged mauve, lilac, or red, inner tube variably pubescent, spotted with scattered black dots, with two yellow folds at the throat. Bracts, bracteoles and calyx densely covered with caducous indumentum of floccose-lanuginose white hairs to glabrescent on surface, and densely floccose-lanuginose on margins. Corolla limb sparsely pubescent-lanuginose on the surface, lanate at the margins.	Sparsely lanuginose just below the inflorescence, glabrous elsewhere.
*C.fissa*	Unequal. Apical calyx lobe considerably shorter than the others or missing entirely. Indumentum white-arachnoid in bud.	Pure white, limb tinged blue-purple, inner tube glabrous, with two yellow folds at the throat. Bracts, bracteoles and calyx villous-arachnoid on the surface and at the margins, corolla lobes densely lanuginose-pubescent.	Hairy to glabrescent.

#### ﻿(ii) *Cistancheviolacea*

We confirm the presence of this species in Israel, and it is easily distinguished from co-occurring taxa because of its purple and white coloration, strong, conspicuous yellow folds in the corolla throat, and lack of indumentum (Table 1). It has been confused in the region with *C.salsa*, probably due to the purple coloration on the corolla limb. In Zohary (1976), despite citing hairy floral parts in the description, the illustration depicts a glabrous plant – probably *C.violacea*. This error was repeated in the Flora Palaestina (Feinbrun-Dothan 1978) and the Analytical Flora of Israel (Feinbrun-Dothan and Danin 1998). We have found *C.violacea* scattered across the Negev, and in sandy areas of the Western Desert, as well as in the Rift Valley in the Arava and the Dead Sea area, and more rarely in the central Negev. It is parasitic on shrubby Amaranthaceae. Hosts observed by us in the region include *Atriplexhalimus*, *Anabasisarticulata* and (less commonly) *Haloxylonnegevensis*; additional possible hosts include *Tamarixnilotica* and *Anabasissetifera*. In some places it co-occurs with *C.tubulosa*, and we have observed rare morphological intermediates in the northern Arava Valley; these are probably hybrids, based on our most recent molecular analysis.

#### ﻿(iii) *Cistanchelaxiflora*

We report the presence of *C.laxiflora* in Israel for the first time. Concomitant with the type species, Israeli populations of *C.laxiflora* have lax spikes of flowers, hairy-based stamens inserted well above the corolla tube base, a more or less regular-campanulate white, lilac-tinged corolla and non-scarious bracts (Beck-Mannagetta 1930). The plants we refer to as *C.laxiflora* are concomitant with the type for this species, with the exception of their 2–4-valved fruits, and their more robust stem and longer inflorescence. Notably, we observed 3–4-valved fruiting capsules in *C.laxiflora*, and occasionally also in *C.tubulosa* and *C.violacea* (Table 1). Fruits with 3–4 valves have not been reported before in species belonging to the traditionally recognised C.sect.Cistanchella (Beck-Mannagetta 1930) nor, in its most recent circumscription, the Widespread Clade (Ataei et al. 2020), all of which are reported to have bivalved fruits. We suspect this feature may have been overlooked due to difficulties in identifying *Cistanche* species in fruit, and a paucity of fruiting material in herbaria. The plants we refer to as *C.laxiflora* are concomitant with the type for this species, with the exception of their 2–4-valved fruits. Molecular data confirms a relationship between our plants sampled in Israel, and *C.laxiflora* collected elsewhere, showing a far broader distribution for this species than was known previously. We note that *C.laxiflora* appears to be virtually indistinguishable from *C.flava*, both of which are originally described from Afghanistan and occur on *Tamarix*; these may be conspecific, in which case the name *C.laxiflora* would take priority.

### ﻿Ecology, distribution and conservation implications

We observed that *C.tubulosa* is widespread in deserts across desertic parts of the country, where it is frequently parasitic on *Atriplexhalimus* and *Tamarix* spp. On the other hand, *Cistancheviolacea* is most frequent along the Rift Valley where it parasitizes *Anabasis* spp., as well as *Atriplexhalimus* on clay-based substrates; it is sometimes covered partially with sand (in the western Negev). Both species are frequent across the deserts of Israel and are not threatened locally. *Cistanchemimii*, *C.tinctoria* and *C.laxiflora* are much more local and are candidates for conservation focus. In the case of *C.mimii*, all currently known populations are restricted to the Negev Highland of Israel, and thus the local threat level for this species is also applicable at a global level.

*Cistanchemimii* is a rare species in Israel, known from fewer than ten localities, predominantly in the Negev Highlands on loess platforms and rocky slopes. At least one population has been eradicated during the expansion of Mitzpe Ramon recently (2019). Most populations do not appear to be threatened by anthropic activity; however, those in the areas of Yeruham and Mitzpe Ramon are particularly sensitive to development. Furthermore, loess plains in the Negev are threatened by agricultural expiation, off-road driving and military activity. The Jordanian population has not been reported for almost a century, so it is impossible to quantify the species’ area of occupancy in the region; however the lack of reports in itself, suggest the plant is also rare there. Therefore, we recommend that *C.mimii* be categorized as Vulnerable according to the IUCN red list criteria, Criterion B1a+b (IUCN, 2012).

*Cistanchelaxiflora* is rare in the region, occurring in four localities, with just a single thriving colony. The largest population of *C.laxiflora* we have observed occurs in a small area around the Ein Saharonim spring, situated on wet alluvial silt-sand and grit, with slightly saline groundwater and in close vicinity of annually flowing spring water. Two other locations nearby the Ein Saharonim population are in Nahal Ardon and Nahal Nekarot’s ‘Horseshoe’; both consist of few individuals. In April 2021, a further population of very few individuals (fewer than 10 observed) was found by D. Ben-Natan in Lower Ein Gidron spring, in the northern Arava Valley. Ein Gidron is a small fresh to saline water spring on sandy clay soil in the extreme desert of the Arava Valley, and is similar to Ein Saharonim in its characteristics and vegetation (dominated by, for example: *T.nilotica*, *A.halimus*, *Nitrariaretusa* (Forssk.) Asch.) Another specimen of *C.laxiflora* was collected by Zohary in Ein Hosb (Ein Hatzeva), another similar spring in the northern Arava Valley, in close vicinity to Ein Gidron (ISRAEL, Arava Valley, Ein Hosb [En-Hatzeva], 9.April.1950., near a spring, on the roots of *Tamarixmaris-mortui* Gutmann, Zohary D. [HUJ123665!]), indicating that populations of *C.laxiflora* formerly occurred in the northern Arava springs. Ein Hosb has since dried due to water abstraction for local agriculture, as have many others in this area; of those populations, the few individuals in Ein Gidron are probably all that remains. The known populations consist of fewer than 200 individuals altogether and should therefore be categorized as Locally Endangered according to the IUCN red list criteria, Criterion D: number of known mature individuals is less than 250. (IUCN, 2012). Makhtesh Ramon is a nature reserve protected by law in Israel, but possible future diminution of the spring’s groundwater level and water flow would further threaten the already precarious local outlook of this species. Ein Gidron, in close proximity to the agricultural field of Hatzeva and Moshav Ein Hatzeva, and is unprotected and disturbed by human activity and by an incursion of the invasive *Acaciasalicina* Lindl. 1838. Furthermore, the aquifers of the northern Arava Valley have been severely diminished in recent decades from over-drawing of the water reserves, causing salination and even desiccation of some of the springs, which is great cause for concern. A fourth population of *C.laxiflora* was discovered by Ron Frumkin in Ein Akrabim, in the lower Nahal Zin valley. Similarly, the species occurs there in sandy silt near a saline spring. Ein Akrabim and the neighbouring Ein Zin are highly threatened by pollution and salination of ground water by mining waste from the neighbouring factory of Oron.

*Cistanchetinctoria* is rare in Israel, and restricted to the southern part of the Arava Valley. The area, especially its sandy and loessial habitats, is highly threatened by agricultural and urban expansion. Although *C.tinctoria* shows an affinity for disturbed habitats at the margins of fields and roadsides, its few populations, in an extremely xeric region, make it a candidate for local conservation.

### ﻿Concluding remarks

*Cistanche* is a taxonomically challenging and poorly understood genus. Like in other holoparasitic genera in the Orobanchaceae, the lack of clearly defined vegetative features can make delineation of species problematic. Here we show that multiple species have been repeatedly misidentified in Israel and the adjoining territories, contributing to confusion in the region. Delineating taxa objectively and reliably will be important for informing conservation priorities in this poorly known genus. Finally, we recommend that extensive field-based examinations across the genus’ range, careful assessment of species’ morphologies and ecologies will be important for informing sampling in further phylogenetic analyses. Extensive sampling and close observation of previously neglected features, together with advanced molecular techniques will be a powerful combined approach to delineating this difficult genus, and informing conservation practice.

### ﻿Key to the *Cistanche* species of Israel:

**Table:** 

1a	Corolla lobes, calyx lobes, bracts and bracteoles floccose-lanuginose on the surface and lanate along the margins. Apical calyx lobe considerably shorter than the others or absent; Bloom emitting a pleasant, citrus-like fragrance	***C.mimii* sp. nov**.
1b	Corolla, bracts, bracteoles and calyx glabrous, calyx lobes subequal	**2**
2a	Bracts, bracteoles and calyx lobes scarious along the margins and serrate. Inflorescence usually dense	**3**
3a	Corolla limb tinged purple or lilac, bracts and bracteoles purple to lilac	**4**
4a	Spike often tall (≤100 cm), robust and dense. Corolla throat yellow, with shallow folds in the throat, limb and external tube tinged lilac or purple. Anthers rounded-elliptic to apiculate	***C.tinctoria***
4b	Spike often shorter (≤80 cm). Corolla tube white inside and out, with large, well-pronounced yellow folds in the corolla throat, limb always conspicuously purple or violet. Anthers acuminate	***C.violacea***
3b	Corolla yellow, lobes occasionally tinged lightly crimson in bud. Bracts and bracteoles whitish to dark grey. Yellow folds in corolla throat shallow and inconspicuous. Anthers rounded-elliptic to apiculate	***C.tubulosa***
2b	Bracts, bracteoles and calyx lobes with non-scarious margins, entire. Corolla white, usually tinged lilac or violet, with two shallow yellow folds in the corolla throat. Filaments inserted at the lower 1/3 of corolla tube; anthers apically rounded, with a small tubercle at the base	***C.laxiflora***

## Supplementary Material

XML Treatment for Cistanche mimii

XML Treatment for Cistanche tubulosa

XML Treatment for Cistanche tinctoria

XML Treatment for Cistanche violacea

XML Treatment for Cistanche laxiflora

## ﻿Additional information

### Use of AI

No use of AI was reported.

### Author ORCIDs

Chris Thorogood https://orcid.org/0000-0002-2822-0182

### Data availability

All of the data that support the findings of this study are available in the main text.
